# *GAL4* Drivers Specific for Type Ib and Type Is Motor Neurons in *Drosophila*

**DOI:** 10.1534/g3.118.200809

**Published:** 2018-12-10

**Authors:** Juan J. Pérez-Moreno, Cahir J. O’Kane

**Affiliations:** Department of Genetics, University of Cambridge, United Kingdom, CB2 3EH

**Keywords:** *Drosophila*, *GAL4*, neuromuscular junction, NMJ, synaptic boutons, glutamatergic synapse, motor neuron

## Abstract

The *Drosophila melanogaster* larval neuromuscular system is extensively used by researchers to study neuronal cell biology, and *Drosophila* glutamatergic motor neurons have become a major model system. There are two main Types of glutamatergic motor neurons, Ib and Is, with different structural and physiological properties at synaptic level at the neuromuscular junction. To generate genetic tools to identify and manipulate motor neurons of each Type, we screened for *GAL4* driver lines for this purpose. Here we describe *GAL4* drivers specific for examples of neurons within each Type, Ib or Is. These drivers showed high expression levels and were expressed in only few motor neurons, making them amenable tools for specific studies of both axonal and synapse biology in identified Type I motor neurons.

*Drosophila* research has contributed for decades to our understanding of both fundamental neuroscience (Bellen *et al.* 2010), and neurological disorders (Ozdowski *et al.* 2015; Tan and Azzam 2017; Xiong and Yu 2018). Much fruitfly neuroscience research is performed at the larval neuromuscular junction (NMJ), a well-characterized system with powerful genetic tools and accessible for physiology and cell biology (Menon *et al.* 2013).

The larval neuromuscular system has a relatively simple pattern that consists, in abdominal hemisegments from A2 to A7, of around 36 motor neurons (MNs) and 30 muscles (Landgraf and Thor 2006; Figure 1), with most muscles co-innervated by more than one Type of MN (Hoang and Chiba 2001; Kim *et al.* 2009). Depending on the NMJ bouton properties, different Types of MN have been described in *Drosophila* larvae. Type I MNs are excitatory and glutamatergic, and are subdivided into Ib (big) and Is (small). Type II and Type III MNs are neuromodulatory, being respectively octopaminergic and peptidergic. In addition, glutamatergic Type I MNs show different muscle innervation patterns: each Type Ib MN typically innervates one muscle, whereas each Type Is MN typically innervates several muscles (Hoang and Chiba 2001; Kim *et al.* 2009). The different Types of Type I MN also differ in their structural and physiological properties at synaptic level (Atwood and Klose 2009). Type Ib synapses show shorter and less extensive branching, and support tonic (sustained) firing, whereas Type Is synapses show more extensive branching, and higher synaptic vesicle release efficacy per impulse, are more phasic (transient), and a higher proportion of their vesicle pool is readily releasable (Atwood *et al.* 1997; Lnenicka and Keshishian 2000; Atwood and Klose 2009; Xing and Wu 2018).

**Figure 1:**
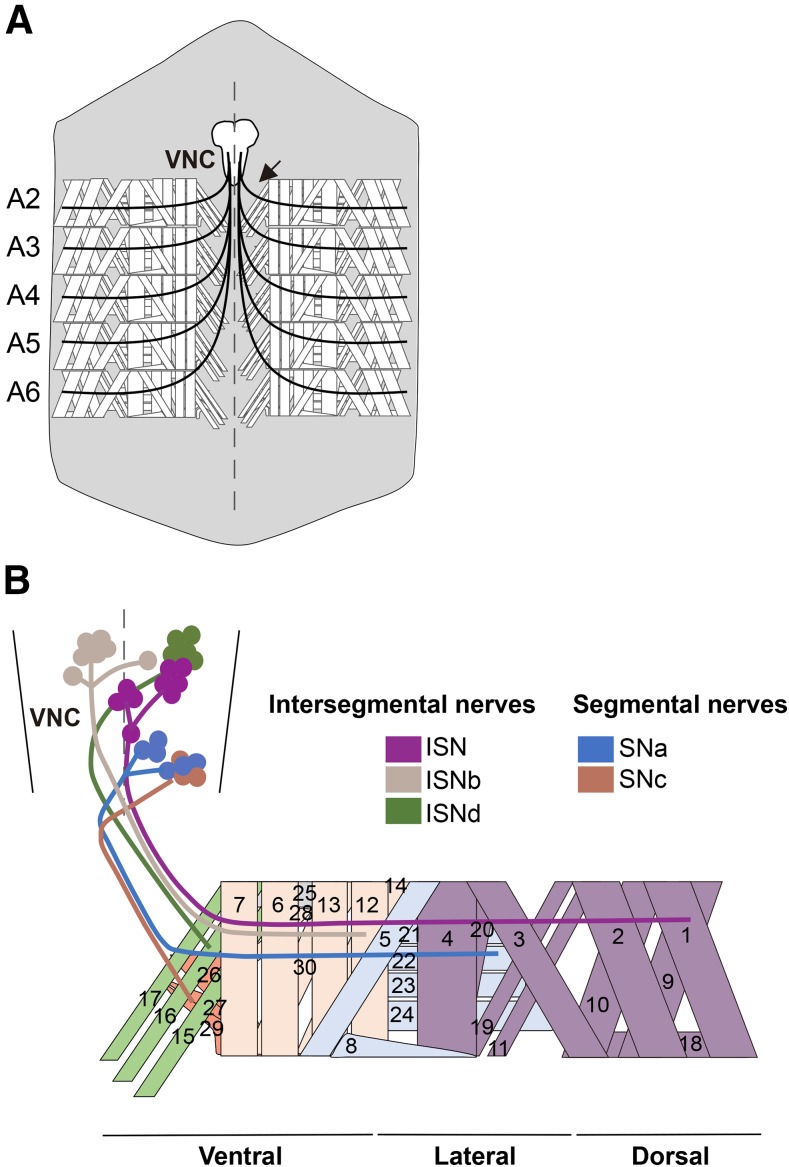
*Drosophila* larval neuromuscular system. A. Scheme of a dissected third instar larva showing the neuromuscular system. Only abdominal segments A2-A6 are represented, whose innervation and muscle pattern are identical. The ventral nerve cord (VNC) consists of segmentally repeated neuromeres that are bilaterally symmetrical across the midline (broken line). Body wall muscles of each hemisegment are innervated by around 30 motor neurons (MNs), whose axons project together from one VNC neuromere, forming a peripheral nerve (black arrow). Anterior is to the top. B. Innervation of one of the abdominal hemisegments shown in A. In the VNC, the MN cell bodies project their axons via six main nerve branches: three intersegmental nerves, two segmental nerves, and a transverse nerve (runs along the segment border but has few MNs, so not represented). The muscles innervated by each nerve branch are represented in a lighter version of the color of each branch, and each muscle number is indicated.

To understand the properties of each Type of NMJ synapse, it is important to identify and manipulate different MN terminals independently. A common approach is labeling (typically using anti-Dlg) of the subsynaptic reticulum (SSR), comprising extensive infolding of the postsynaptic cell membrane, and whose amount differs among MN Types (Zito *et al.* 1999; Menon *et al.* 2013). However, this approach has several limitations, especially when trying to distinguish different MNs with overlapping branches at the same NMJ: fewer channels available for fluorescence microscopy, especially in live imaging, and potential misidentification of bouton Types in genotypes or environmental conditions that affect SSR or bouton size. An approach to avoid all these limitations would be to use markers based on the genetic identity of the MN.

Using the *GAL4/UAS* system, is possible to express markers or functional proteins specifically in those cells expressing *GAL4* (Brand and Perrimon 1993). While neuromodulatory MNs (Types II and III) are not as extensively studied as excitatory glutamatergic MNs (Ib and Is), specific *GAL4* drivers have been reported for Type II (Cole *et al.* 2005; Stocker *et al.* 2018) and Type III (Park *et al.* 2003; Vömel and Wegener 2007; Koon and Budnik 2012) MNs. Several useful *GAL4* drivers are expressed in Ib and Is MNs, but in some cases they are also expressed in neuromodulatory MNs (Koon and Budnik 2012), or in both Type Ib and Is MNs (Fujioka *et al.* 2003). Other Type I-specific drivers are steroid-activated (Nicholson *et al.* 2008). In addition, most of the mentioned lines are expressed in multiple MNs, and are therefore less amenable for studies on identifiable axons for which labeling of no more than 2 or 3 MNs would be desirable.

We therefore aimed to identify *GAL4* drivers specific for small numbers of Type Ib or Is MNs. For this, we screened expression patterns in the larval abdominal nerve cord, in some of the neuronal *GAL4* lines generated by the FlyLight project (https://www.janelia.org/project-team/flylight), and identified two glutamatergic *GAL4* lines, one specific for a single Type Ib MN, and the other specific for two Type Is MNs. We also identified other potential drivers for neuromodulatory MNs (Type II/III). We propose the two Type I-specific lines as tools of general interest for the *Drosophila* neuroscience community, improving the rigor and the accuracy of the study of both axonal and presynaptic biology.

## Materials and Methods

### Drosophila genetics

All *Drosophila* stocks were obtained from the Bloomington *Drosophila* Stock Center, and are listed in Table 1.

**Table 1:** *Drosophila* stocks used in this work. References: 1 (Pfeiffer *et al.* 2008; Jenett *et al.* 2012); 2 (Han and Jan 2011); 3 (Summerville *et al.* 2016)

Genotype	RRID	Reference
*w^1118^* ;; *GMR24H01-GAL4*	BDSC_48054	1
*w^1118^* ;; *GMR26B02-GAL4*	BDSC_49321	1
*w^1118^* ;; *GMR27E09-GAL4*	BDSC_49227	1
*w^1118^* ;; *GMR29H05-GAL4*	BDSC_48094	1
*w^1118^* ;; *GMR31C03-GAL4*	BDSC_48103	1
*w^1118^* ;; *GMR35F03-GAL4*	BDSC_49914	1
*w^1118^* ;; *GMR43G02-GAL4*	BDSC_49555	1
*w^1118^* ;; *GMR45A05-GAL4*	BDSC_50218	1
*w^1118^* ;; *GMR56G03-GAL4*	BDSC_46336	1
*w^1118^* ;; *GMR64B05-GAL4*	BDSC_39292	1
*w^1118^* ;; *GMR65H09-GAL4*	BDSC_47389	1
*w^1118^* ;; *GMR69G08-GAL4*	BDSC_46617	1
*w^1118^* ;; *GMR74A06-GAL4*	BDSC_47398	1
*w^1118^* ;; *GMR80C02-GAL4*	BDSC_47055	1
*w^1118^* ;; *GMR84D10-GAL4*	BDSC_40392	1
*w^1118^* ;; *GMR85F10-GAL4*	BDSC_40434	1
*w^1118^* ;; *GMR91E03-GAL4*	BDSC_48631	1
*w^1118^* ;; *GMR92C02-GAL4*	BDSC_47190	1
*w^1118^* ;; *GMR94G06-GAL4*	BDSC_40701	1
*w^1118^* ;; *UAS-CD4*::*tdGFP*	BDSC_35836	2
y^1^ w* ; *UAS-CD4*::*tdGFP*	BDSC_35839	2
*w^1118^* ; *UAS-tdTomato*::*Sec61β*	BDSC_64746	3

### Histology and immunomicroscopy

Third instar larvae were dissected in chilled Ca^2+^-free HL3 solution (Stewart *et al.* 1994), and fixed for 15 min in PBS with 4% formaldehyde. For immunostaining, the dissected preparations were permeabilized in PBS containing 0.1% Triton X-100 (PBT) at room temperature for 1 h. F-actin was stained by incubating dissected samples for 30 min at room temperature with Texas Red X-Phalloidin 1:400 (T7471, Thermo Fisher Scientific). For immunostaining, after permeabilization, samples were blocked in PBT with 4% bovine serum albumin for 30 min at room temperature, incubated with primary antibodies overnight at 4°, and finally incubated with secondary antibodies for 2 h at room temperature. Primary antibody was: mouse anti-Dlg 1:100 (4F3, Developmental Studies Hybridoma Bank; Parnas *et al.* 2001), and secondary antibody was: goat anti-mouse conjugated to Alexa-647 (A21247, Thermo Fisher Scientific). Visualization of CD4::GFP and tdTomato::Sec61β markers was performed via direct imaging, without immunostaining. Processed preparations were mounted in Vectashield (Vector Laboratories), and images were collected using EZ-C1 acquisition software (Nikon) on a Nikon Eclipse C1si confocal microscope (Nikon Instruments, UK). Images were captured using a 40x/1.3NA oil objective.

### Image analysis and figure preparation

All the microscopy images shown are maximum intensity projections derived from confocal stacks. In the VNC, labeled axonal projections were tracked through sections from cell bodies toward the peripheral nerve, and from the peripheral nerve to the NMJ and muscles. In Figure 6 and 9, outline of muscles in specific NMJs was identified by using bright-field microscopy. Similarly, the innervation pattern presented in Supp. Table S1 was identified by using bright-field microscopy. All images were opened, analyzed and processed using ImageJ FIJI (https://fiji.sc) (Schindelin *et al.* 2012). Figures were made using Adobe Illustrator.

### Reagent and data availability

Reporters and FlyLight Project *GAL4* lines used in this study are available from the Bloomington *Drosophila* Stock Center (Table 1). The SuppData_Legends.pdf file contains detailed descriptions of all supplemental files. The SuppFig1.pdf file shows genomic maps of the regions that contain the fragments that control expression in *GMR27E09* and *GMR96G06* lines. The SuppTableS1.pdf file contains a summary of the collected data for the different GAL4 drivers screened. Files SuppMovieS1.mp4 to SuppMovieS4.mp4 contain confocal 3D projections of MN cell bodies and adjacent axonal regions in the VNC for *GMR27E09* (plasma membrane reporter in file SuppMovieS1.mp4; ER reporter in file SuppMovieS2.mp4) and *GMR94G06* (plasma membrane reporter in file SuppMovieS3.mp4; ER reporter in file SuppMovieS4.mp4). The underpinning dataset for this paper is available at the University of Cambridge Repository (https://www.repository.cam.ac.uk): https://doi.org/10.17863/CAM.33651. Supplemental material is available at Figshare: https://doi.org/10.25387/g3.7423889.

## Results

### Screening for potential drivers specific for glutamatergic MNs

The FlyLight project has generated around 7,000 transgenic *Drosophila* lines, in each of which expression of *GAL4* is controlled by a different transcriptional enhancer that often drives expression in small subsets of neurons (Pfeiffer *et al.* 2008; Jenett *et al.* 2012). To identify drivers specific for different classes of MN, we first reviewed images of the larval central nervous system, for 418 *GAL4* lines listed as driving expression of the *UAS-mCD8*::*GFP* reporter in the abdominal ventral nerve cord (http://flweb.janelia.org/cgi-bin/flew.cgi). We prioritized candidates using several criteria: single or as few as possible cell bodies per neuromere in the VNC; axons visible in the nerves that innervate the body wall musculature (peripheral nerves); moderate or high GFP levels in axons.

**Figure 2:**
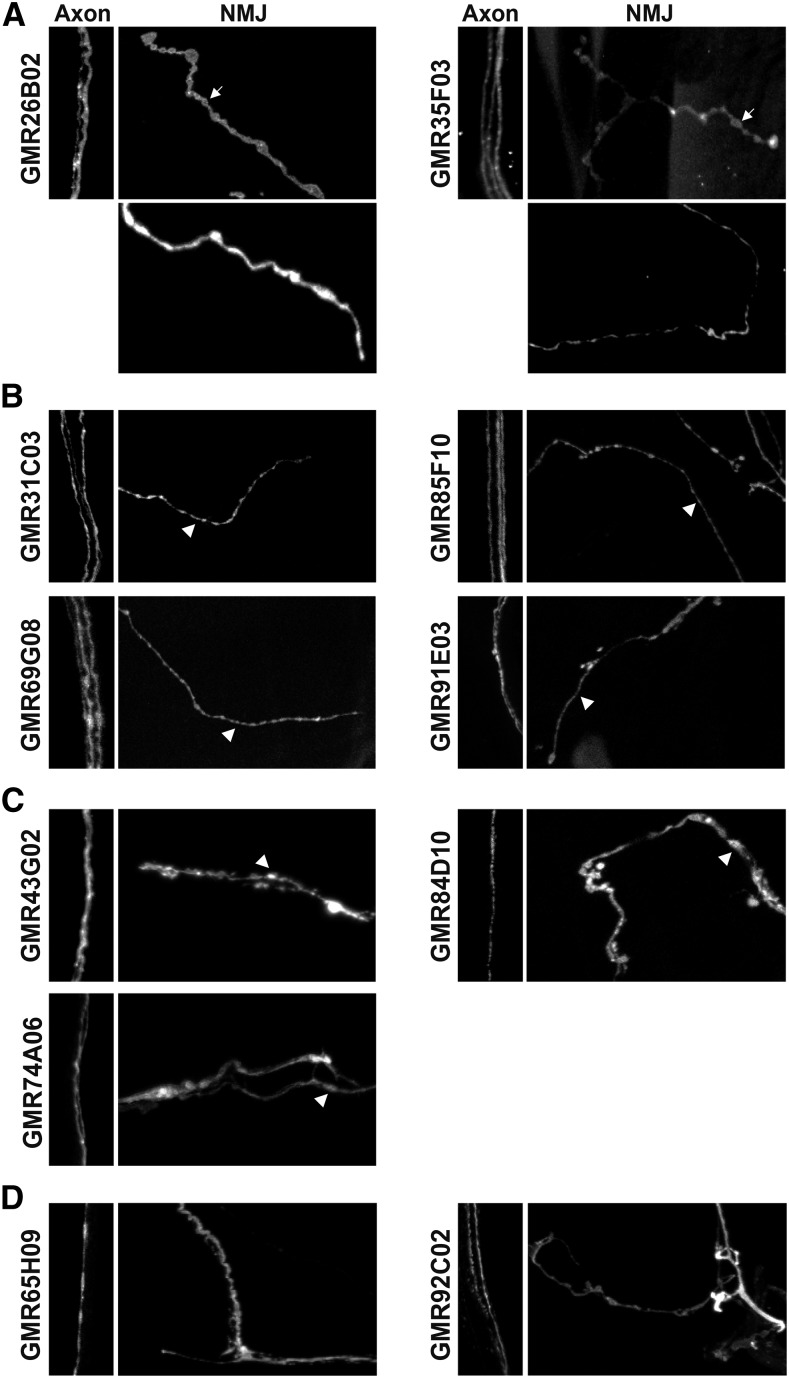
Examples of different MN Types recovered in screen. Confocal projections showing plasma membrane marker (CD4::GFP) in larvae expressing *UAS-CD4*::*GFP* and *UAS-tdTom*::*Sec61β* under the control of different *GAL4* drivers. Axon images show a region of the peripheral nerve where the MNs expressing the reporter can often be distinguished individually, and NMJ images show representative examples of the Types of presynaptic terminal identified. Axon panels: 15x40 μm; NMJ panels: 60x40 μm. A. Lines potentially expressed in both Type I (top NMJ panels), which show short presynaptic branches with large presynaptic boutons (arrows) (Atwood and Klose 2009), and neuromodulatory MNs (bottom NMJ panels; Type III and Type II respectively in *GMR26B02* and *GMR35F03*). B. Lines potentially expressed in Type II MNs, which show long NMJ branches and small presynaptic boutons (arrowheads) (Atwood and Klose 2009). C. Lines potentially expressed in Type III MNs, which innervate only a few muscles between the ventral and lateral regions of the hemisegment (Supp. Table S1), and present elliptical-like shaped presynaptic boutons (arrowheads) (Jia *et al.* 1993; Atwood and Klose 2009). D. Lines that express in an unknown neuromodulatory MN Type (Type II or Type III) but not in Type I MNs.

**Figure 3:**
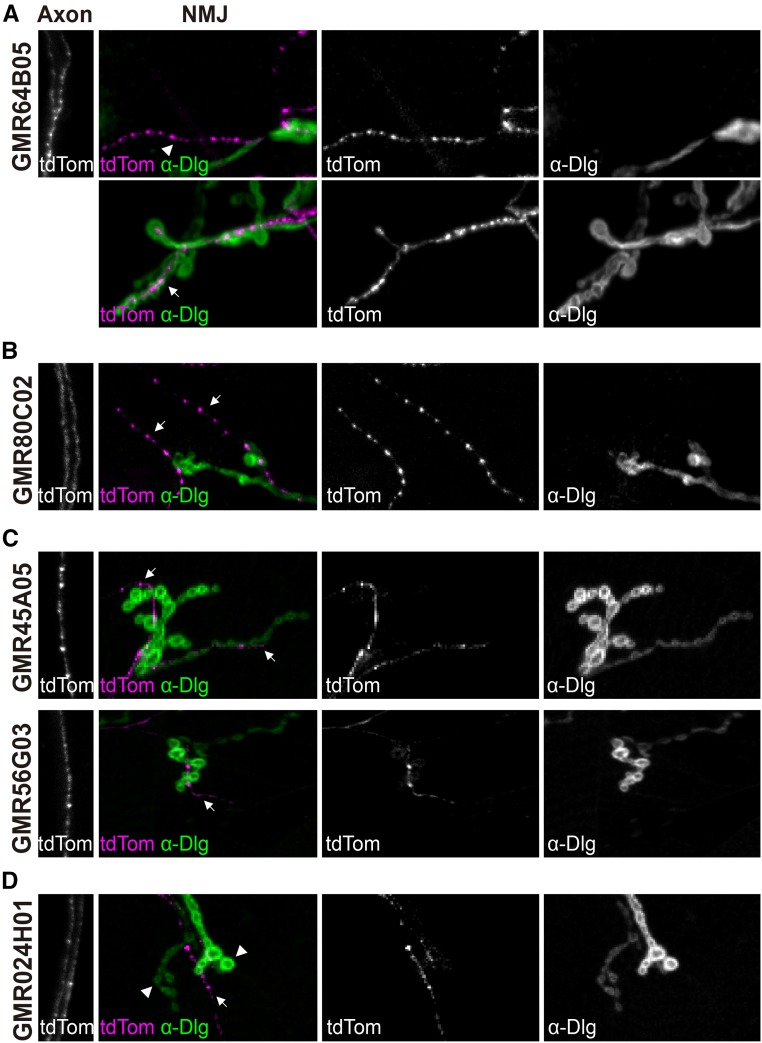
Use of anti-Dlg to confirm MN Types. Confocal projections of larvae expressing *UAS-tdTom*::*Sec61β* under the control of different *GAL4* drivers, organized as in Figure 2. Immunostaining against Dlg protein helps distinguish between different Type of boutons. A. Line potentially expressed in Type II MNs (top NMJ panel; arrowhead), and in Type Ib (bottom NMJ panel; arrow), which show high Dlg signal (Menon *et al.* 2013). B. Line potentially expressed in Type II MNs, which show long NMJ branches (Atwood and Klose 2009) and no Dlg signal (arrows) (Menon *et al.* 2013). C. Lines potentially expressed in Type III MNs, which innervate only a few muscles between the ventral and lateral regions of the hemisegment (Supp. Table S1) (Atwood and Klose 2009), and no Dlg signal (arrows) (Menon *et al.* 2013). D. Line that expresses in an unknown neuromodulatory MN-Type (arrow) but not in Type I MNs (arrowheads).

We then analyzed selected candidate lines (Table 1), using two different reporters to verify the *GAL4* expression levels and distribution: a plasma membrane marker (*UAS-CD4*::*GFP*) to visualize the whole neuron, including cell body, axonal and presynaptic regions; and an endoplasmic reticulum (ER) marker (*UAS-tdTom*::*Sec61β*), previously described as continuously distributed throughout the whole neuron (Summerville *et al.* 2016; Wu *et al.* 2017). Unless otherwise specified, we refer below to the plasma membrane marker. In addition, we checked the Type of NMJ produced by such cells. Type II presynaptic terminals are smaller and show longer branch length than Type I terminals, while Type III MNs show characteristic elongated or elliptical presynaptic terminals with an intermediate size between Type I and II (*Jia et al.* 1993). Therefore, we used these properties to choose potential glutamatergic *GAL4* lines (Figure 2), and additionally used anti-Dlg labeling on a subset of lines (Figure 3) to assess the robustness of our identification criteria. We stopped screening once we found a line expressing specifically in either Type Is or Type Ib MNs, *GMR27E09* and *GMR94G06* respectively.

### GMR27E09-GAL4 drives expression in two Type Is MNs per hemisegment

**Figure 4:**
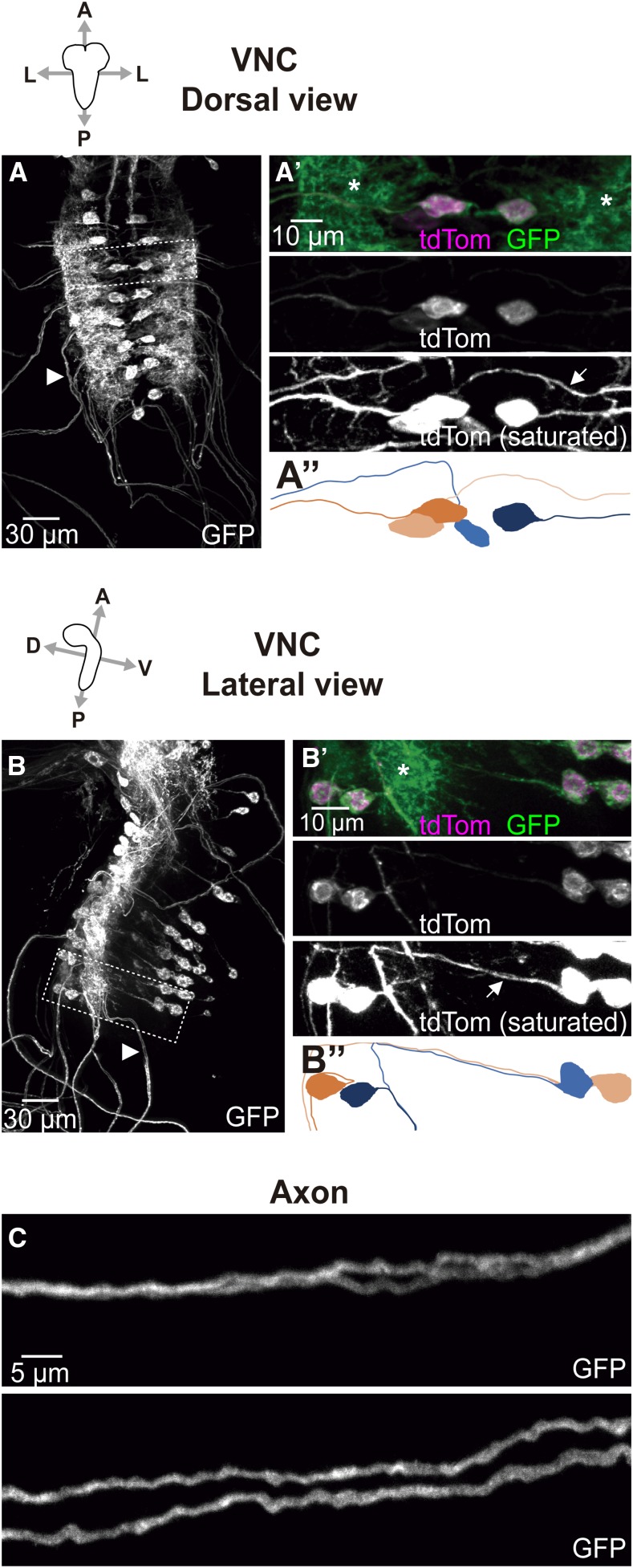
*GMR27E09-GAL4* expression in VNC. Dorsal (A) and lateral (B) confocal projections showing VNC of larvae expressing *UAS-CD4*::*GFP* and *UAS-tdTom*::*Sec61β* under control of *GMR27E09-GAL4*. A’ and B’ show magnification of the areas indicated with a broken line in A and B respectively. Plasma membrane marker (CD4::GFP) reveals expression of *GMR27E09-GAL4* in two motor neuron cell bodies per hemineuromere (A’). Unlike plasma membrane marker, which shows high levels in neuropil (asterisks), axonal projection of each cell body can be easily tracked (arrows) using the ER marker (*tdTom*::*Sec61β*), which is preferentially distributed in cell bodies and axons. The corresponding representations are shown in A’’ and B’’, where neurons with cell bodies in different hemineuromeres are represented in orange and blue. Light and dark versions of the colors are used to distinguish between the two neurons within each hemineuromere. Examples of their axonal projections into the same peripheral nerve are indicated by arrowheads in A and B. A, anterior; P, posterior; L, lateral; D, dorsal; V, ventral. C. In each peripheral nerve, plasma membrane signal reveals regions where both axons mostly overlapped (top panel), or remained apart (bottom panel). Both examples shown in C correspond to different parts of the same peripheral nerve. Anterior and posterior regions are to the left and to the right respectively.

**Figure 5:**
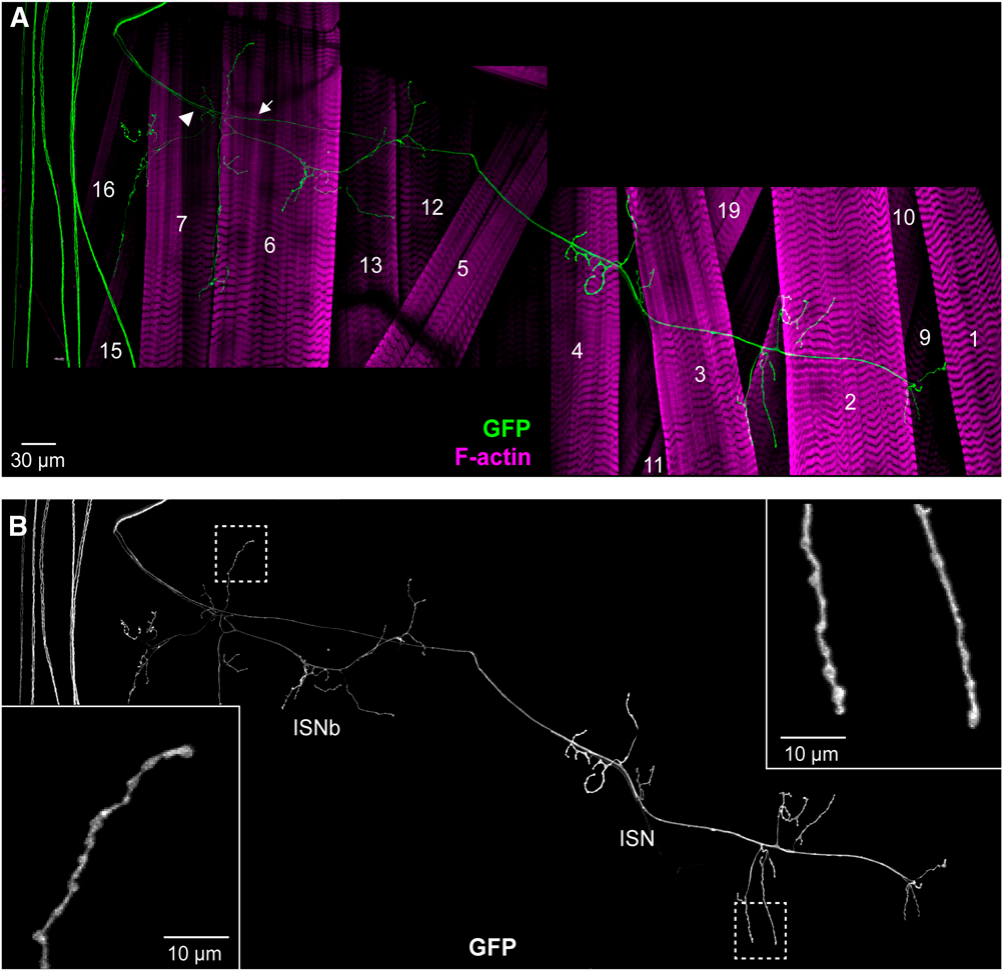
Muscle innervation by MNs expressing *GMR27E09-GAL4*. Composite of several confocal projections showing the NMJ of a whole abdominal hemisegment in a *GMR27E09-GAL4/UAS-CD4*::*GFP* larva. A. Roots of ISNb and ISN nerve branches are indicated by an arrowhead and an arrow respectively. Phalloidin staining of F-actin highlights muscle cells. Recognizable muscles are indicated with their corresponding number. B. Single channel image of (A) showing GFP expression. Magnifications of the areas indicated with a broken line are shown in the bottom left and in the top right corners. Midline is on the left; anterior is to the top.

*GMR27E09-GAL4* showed expression in two prominent cell bodies per hemineuromere (Figure 4 A, B). These were located close to the midline, and projected their axons toward each peripheral nerve, one ipsilaterally and one contralaterally to the cell body (Figure 4 A-B’’). One of these cell bodies (projecting ipsilaterally) was located in the dorsal region of the VNC, and the other (projecting contralaterally) in the ventral region (Figure 4 B-B’’; Supp. Movies S1, S2). In the peripheral nerve, where both axons run parallel to each other, their paths were too close to distinguish by confocal microscopy in some regions (top panel on Figure 4 C), but we frequently found regions where both axons could be easily distinguished (bottom panel on Figure 4 C); this did not obviously correlate with proximodistal position along the peripheral nerve. Each axon innervated several internal muscles from a nerve branch found close to the intersegmental region. One of the MNs innervated proximal muscles (ventral and lateral), while the other MN innervated distal muscles (lateral and dorsal) (Figure 5 A, B). Based on these data, we conclude these MNs are respectively part of the ISNb and ISN branches of the intersegmental nerve (Hoang and Chiba 2001; Figure 1).

**Figure 6:**
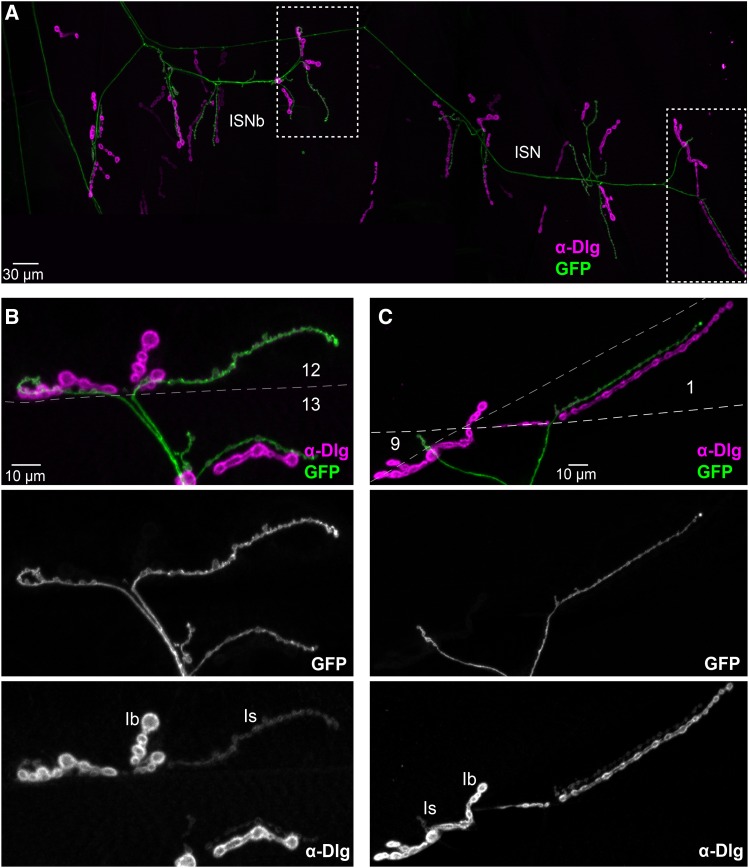
*GMR27E09-GAL4* is expressed in two Type Is MNs. A. Composite of several confocal projections showing the NMJ of a whole abdominal hemisegment in a *UAS-CD4*::*GFP*, *UAS-tdTom*::*Sec61β/+* ; *GMR27E09-GAL4/+* larva. Immunostaining of Dlg helps distinguish between Type Ib and Type Is boutons. Midline is on the left; anterior is to the top; positions of major muscles can be inferred by comparison to Fig. 3. Areas inside broken lines are shown at higher magnification in (B) and (C). Magnified views of NMJs from ISNb nerve on muscles 12/13 (B), and ISN nerve on muscles 1/9 (C), show that MNs expressing *GMR27E09-GAL4* produce only Is-Type boutons. Broken lines indicate muscle outlines. In C, where both innervated muscles overlapped, the edge of muscle 9 is indicated by a gray broken line, while edge of muscle 1 is indicated by a thick white broken line. Examples of Ib and Is boutons are indicated in the anti-Dlg channel.

The short length of the presynaptic branches and the relatively big size of the presynaptic boutons, suggested that the two MNs expressing *GMR27E09-GAL4* could be Type I and glutamatergic (Figure 5 A, B). To test this possibility, and also distinguish between Type Ib and Is glutamatergic terminals, we double-labeled for *GAL4*-dependent reporter expression and anti-Dlg, whose postsynaptic distribution shows different sizes and levels between Ib and Is boutons (Menon *et al.* 2013). The NMJ presynaptic terminals of both labeled axons showed detectable levels of Dlg, but did not include Type Ib boutons that showed the biggest and brightest anti-Dlg signals (Figure 6 A-C**)**. Since Dlg is absent in Type II and III NMJs (Menon *et al.* 2013), we conclude that both MNs expressing *GMR27E09-GAL4* are of Type Is.

At least three Type Is MNs have been described in larval abdominal hemisegments, each innervating multiple muscles from the ISN, SNa and SNb/d branches (Hoang and Chiba 2001; Kim *et al.* 2009). Our data suggest that *GMR27E09-GAL4* line is expressed in two of these: ISNb/d-Is (also known as RP5; Mauss *et al.* 2009), which innervates ventral musculature contralaterally; and ISN-Is (also known as RP2; Landgraf *et al.* 2003b), which innervates lateral and dorsal musculature ipsilaterally (Hoang and Chiba 2001; Kim *et al.* 2009).

### GMR94G06-GAL4 drives expression in a single Type Ib MN per hemisegment

**Figure 7:**
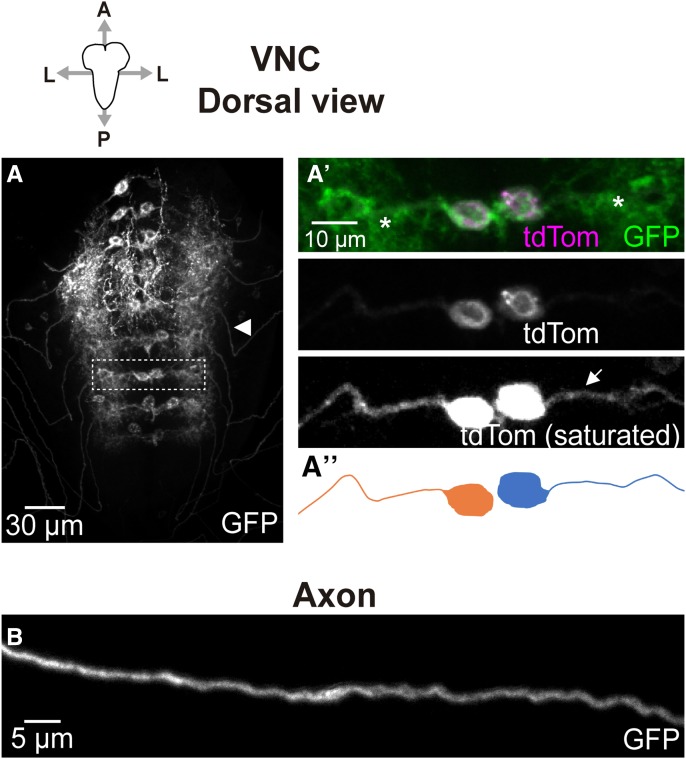
*GMR94G06-GAL4* expression in VNC. A. Dorsal confocal projections showing VNC in a *UAS-CD4*::*GFP*, *UAS-tdTom*::*Sec61β/+* ; *GMR94G06-GAL4/+* larva, where plasma membrane marker (CD4::GFP) reveals expression of *GMR94G06-GAL4* in two cell bodies of muscle-innervating neurons per neuromere. A’. Magnification of the area indicated with a broken line in A. The axonal projection (arrow) of each cell body can be tracked using ER marker (*tdTom*::*Sec61β*), which, in contrast to the GFP plasma membrane marker, is not obscured by high signal levels in the neuropil (asterisks). The corresponding representation is shown in A’’, where neurons innervating different hemisegments are represented in orange and blue. A, anterior; P, posterior; L, lateral. B. In the peripheral nerve, plasma membrane signal reveals a single axon. Anterior and posterior regions are on the left and the right respectively.

**Figure 8:**
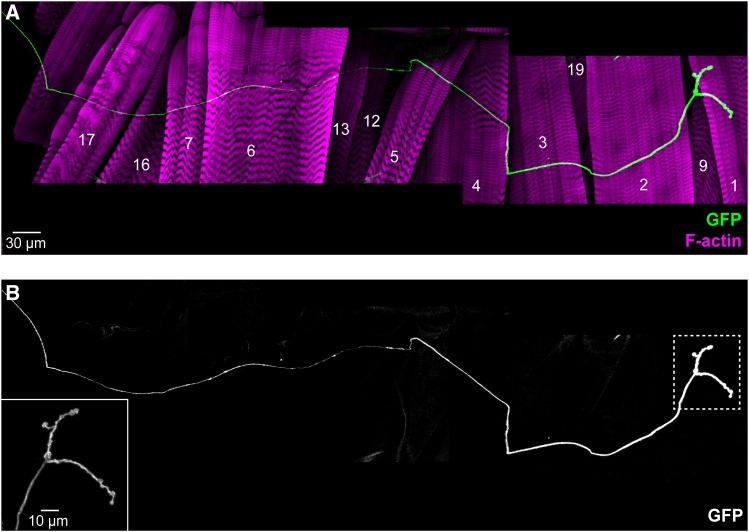
Muscle innervation by MNs expressing *GMR94G06-GAL4*. A. Composite of several confocal projections showing the NMJ of a whole abdominal hemisegment in a *GMR94G06-GAL4/UAS-CD4*::*GFP* larva. Phalloidin staining of F-actin highlights muscle cells. Recognizable muscles are indicated with the corresponding number. B. Single channel image of (A) showing GFP expression. Magnification of the area indicated with a broken line is shown in the bottom left corner. Midline is on the left; anterior is to the top.

Line *GMR94G06-GAL4* was expressed in a single prominent cell body per hemineuromere in the VNC (Figure 7 A). This was located at the dorsal region of the VNC close to the midline, and projected its axon ipsilaterally toward the peripheral nerve (Figure 7 A’, A’’; Supp. Movies S3, S4). Therefore, each peripheral nerve contained just a single axon (Figure 7 B). This axon innervated a dorsal muscle (muscle 1) from a nerve branch found close to the intersegmental region (Figure 8 A, B). Therefore, we conclude that the axon follows the ISN branch of the intersegmental nerve (Figure 1). No other innervation of the body wall muscles was detected (Figure 8 A, B).

**Figure 9:**
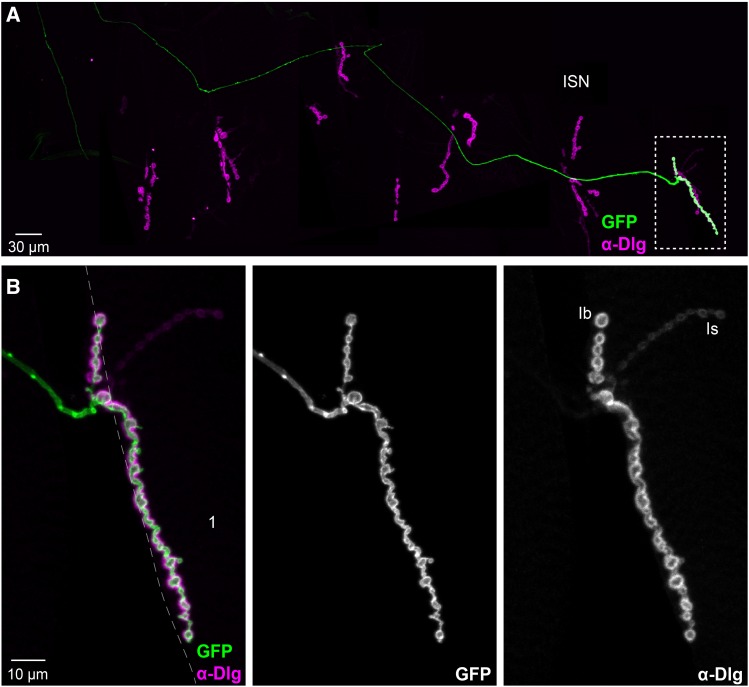
*GMR94G06-GAL4* is expressed in a single Type Ib MN. A. Composite of several confocal images showing the NMJ of a whole abdominal hemisegment in a *UAS-CD4*::*GFP*, *UAS-tdTom*::*Sec61β/+* ; *GMR94G06-GAL4/+* larva. Immunostaining against Dlg protein helps distinguish between Type Ib and Type Is boutons. Midline is on the left; anterior is to the top. Area inside broken lines is shown at higher magnification in (B). Magnified view of NMJ on muscle 1 (B) shows that MN expressing *GMR94G06-GAL4* only produces Ib-Type boutons on muscle 1. Broken line indicates muscle outlines. Examples of Ib and Is boutons are indicated in the anti-Dlg channel.

The large size of the presynaptic boutons, the short length of the NMJ branches, and innervation of a single muscle, suggest that the MN expressing *GMR94G06-GAL4* is a Type Ib glutamatergic neuron. We confirmed this by showing that *GMR94G06-GAL4* drove reporter expression only in presynaptic boutons with high levels of Dlg (Figure 9 A, B). Based on the cell body position, the innervation of muscle 1, and the Type of NMJ (Ib), we conclude that *GMR94G06-GAL4* is specifically expressed in MN1-Ib (also known as aCC) (Hoang and Chiba 2001; Kim *et al.* 2009).

### Regulatory regions of GMR27E09-GAL4 and GMR94G06-GAL4 drivers

The MN-specific expression patterns are regulated by enhancers that are used to drive *GAL4* expression (Pfeiffer *et al.* 2008; Jenett *et al.* 2012). The driver *GMR27E09-GAL4*, expresses *GAL4* using a fragment mainly from one of the introns of the *Fmr1* gene, which is present in all recorded transcripts of *Fmr1* (Supp. Fig. S1 A). In *GMR94G06-GAL4*, the fragment controlling *GAL4* expression comes from an intergenic region between two genes of the same family, *dpr4* and *dpr5* (Supp. Fig. S1 B).

### Drivers for other MN Types

In addition, we found several lines expressing in either Type II or Type III MNs, as well as three lines potentially expressing both in excitatory Type I MNs, and either Type II or Type III modulatory motor neurons (Figure 2; Figure 3; Supp. Table S1).

Some of these lines could be interesting for other studies, since they are expressed in single MNs. First, *GMR56G03*, *GMR84D10* and *GMR45A05* were potentially expressed in the same MN, according to the VNC expression data from Janelia (http://flweb.janelia.org/cgi-bin/flew.cgi); of these, *GMR45A05* showed high expression levels in this MN and was not highly expressed in other tissues (Supp. Table S1). This MN innervates only a few muscles between the ventral and lateral regions of the hemisegment, with elliptical shaped presynaptic boutons (Figure 2 and Figure 3). Therefore, it may be a Type III MN (Jia *et al.* 1993; Atwood and Klose 2009). Since only one Type III MN has been identified (Vömel and Wegener 2007), these drivers may be expressed in it, like the previously described *CCAP-GAL4* (Park *et al.* 2003) or *20C11-GAL4* (Koon and Budnik 2012). Second, *GMR65H09* line is expressed in an MN included in the transversal nerve (Supp. Table S1), making it a potentially interesting driver to study neurons that traverse this poorly characterized nerve.

## Discussion

### Importance of the identification of GMR27E09-GAL4 and GMR94G06-GAL4 drivers

We have identified and characterized two *GAL4* lines specific for different Types of *Drosophila* larval glutamatergic MNs. *GMR27E09-GAL4* is expressed in MN ISNb/d-Is (RP5) and MN ISN-Is (RP2), while *GMR94G06-GAL4* is expressed in MN1-Ib (aCC). Interestingly, one of the most widely used drivers to analyze specific glutamatergic NMJs is the line *RN2-GAL4* (or *eve-GAL4*), which is expressed in aCC and RP2 (Fujioka *et al.* 2003; Landgraf *et al.* 2003a). Therefore, the drivers identified here allow us to study these same well characterized MNs, aCC and RP2, but separately from each other. The alternative approach of clonal labeling of individual neurons (Roy *et al.* 2007) requires complex genetics and is not consistent between samples. Here we identified classical *GAL4* drivers expressed only in Type Is or Type Ib MNs, which are respectively expressed in two MNs or a single MN per hemisegment, allowing simultaneous axonal and NMJ studies in both fixed or *in vivo* experiments.

### MN identity and regulation of GMR27E09-GAL4 and GMR94G06-GAL4 drivers

The gene expression patterns that govern identity of each MN including its pathfinding and synaptic partners (Landgraf and Thor 2006) are ultimately regulated by enhancers. The *GMR27E09-GAL4* and *GMR94G06-GAL4* drivers express *GAL4* under the control of genomic regulatory regions from near the *Fmr1*, and *dpr4* or *dpr5* genes respectively.

*Fmr1* encodes an RNA-binding protein, which acts as a neural growth brake regulating RNA trafficking, translation and neuronal excitability, and whose downregulation contributes to Fragile X syndrome in humans (Banerjee *et al.*, 2010). Although *Fmr1* is widely expressed (Wan *et al.*, 2000) and its function is generally required in *Drosophila* larvae MNs (Zhang *et al.*, 2001), the regulatory sequence controlling *GMR27E09-GAL4* (Supp. Fig. S1 A) drives much more restricted expression than that of *Fmr1*.

The *Dpr* family comprises 21 different genes, which encode neuronal surface receptors required for synapse organization. Several *Dpr* genes are expressed in different subsets of neurons in the *Drosophila* larval VNC, acting as synaptic labels and thus allowing specific synaptic connectivity (Carrillo *et al.* 2015). Although there is no information available about the expression patterns of the *Dpr4* and *Dpr5* genes located close to the regulatory region in *GMR94G06-GAL4* (Supp. Fig. S1 B), it is not unexpected that this regulatory region controls expression in a specific MN. During the preparation of this manuscript another study in parallel characterized a *GAL4* driver specific for Type Is MNs, *DIP-α-T2A-GAL4* (Ashley *et al.* 2018). As *GMR27E09-GAL4*, this driver is expressed in two of the three existing Type Is MNs, and interestingly, its expression is controlled by a genomic region from *DlP-α*, which encodes a Dpr-binding protein.

### Future perspectives

*GMR27E09-GAL4* and *GMR94G06-GAL4* are specific drivers for two Type Is MNs and a single Type Ib MN, respectively, per *Drosophila* larvae hemisegment, thus allowing the specific labeling of these Types of MN. This will allow labeling, live imaging, and manipulation of these specific classes of MN, to better understand the biology of the NMJ and its physiologically diverse Types of synapse.
